# Chemical composition and growth characteristics of *Amorphophallus bulbifer*

**DOI:** 10.1186/s12870-025-06462-5

**Published:** 2025-04-10

**Authors:** Jinwei Li, Huifang Yuan, Yanxiong Gong, Susu Xu, Minghui Chen, Zhu Cun, Yanwei Ruan, Xiangshuai Yan

**Affiliations:** 1 Yunnan Institute of Tropical Crops, Jinghong, China; 2 College of Agronomy and Biotechnology, Yunnan Agricultural University, Kunming, China

**Keywords:** *Amorphophallus bulbifer*, Growth, Yield, Quality, Correlation analysis, Principal components analysis (PCA), Metabolome

## Abstract

Konjac is an important horticultural vegetable and characteristic cash crop. *Amorphophallus bulbifer* has many advantages such as high yield, good quality and strong resistance. However, the awareness of collection and classification of germplasm resources is not strong, the germplasm resources are mixed in planting, there is a lack of good varieties, and the chemical composition of *A. bulbifer* is still unclear. Therefore, 5 self-selected *A. bulbifer* germplasm resources were used as experimental materials to study the growth, physicochemical properties and nutritional components by correlation analysis, principal components analysis (PCA) and metabolomic analysis. The results showed that *A. bulbifer* had strong growth adaptability, konjac glucomanan (KGM) content was 34.88% to 65.72%, viscosity was up to 19513.33 mPa.s, in addition, it was rich in starch, crude protein, amino acids and other nutrients. The aboveground growth and underground yield of konjac had significant positive correlation with each other (*p*< 0.05) and KGM had significant positive correlation with viscosity (*r* = 0.8, *p*< 0.001), but had significant negative correlation with starch, soluble sugar and crude fiber (*p*< 0.01), respectively. 945 metabolites were detected in the metabolome. Besides the primary metabolites, there were also abundant secondary metabolites such as flavonoids, alkaloids, organoheterocyclic compounds, phytohormones and other secondary metabolites. Carbohydrates and its derivatives, amino acids and its derivatives, organic acids and its derivatives, organoheterocyclic compounds and lipids were the main differential metabolites, among which D-Gluconic acid was an important differential metabolite. In conclusion, this study confirmed that *A. bulbifer* has good physical and chemical properties and rich nutritional components, suitable for the development and utilization of high value-added products.

## Highlights

1. There was a significant positive correlation among petiole diameter, plant height, leaf width and yield, and the correlation coefficient was 0.59 or above.

2. Konjac glucomannan was positively correlated with viscosity and negatively correlated with starch, soluble sugar and crude fiber.

3. By principal components analysis, 5 *Amorphophallus bulbifer* germplasm resources were divided into 3 categories. The first category was ‘YunRe1701’, which was significantly higher than other varieties and had the highest yield. The second category was ‘YunRe1709’ and ‘YunRe1726’ with KGM content of about 65%. The third category was ‘YunRe1707’ and ‘YunRe1710’ with better comprehensive performance.

4. D-gluconic acid was an important differential metabolite among *A. bulbifer* varieties.

## Introduction

Konjac is a perennial herbaceous plant belonging to the genus *Amorphophallus* in the family Araceae, which mainly originated in Indochina Peninsula and southern Yunnan in China, and its underground corms are rich in konjac glucomanan (KGM) [1]. KGM is a soluble dietary fiber, which can maintain the normal physiological activities of intestinal cells, promote the proliferation and metabolism of beneficial bacteria in the intestine, reduce the mutation of cancer cells and maintain intestinal health [2, 3]; improve glucose metabolism and reduce blood sugar, blood pressure and cholesterol [4]; promote intestinal peristalsis, lose weight and improve constipation [5]; the composite membrane prepared by KGM has better permeability, can slow down the loss of water and the decrease of hardness in the package, has better fresh-keeping effect than PE membrane, and is degradable, which contributes to the protection of the ecological environment [6], and plays an important role in the fields of medical treatment, food, industry and so on [7].

*Amorphophallus bulbifer* is a general term for konjac with bulbils (aerial corms) on its leaves, which is mainly distributed in tropical and subtropical regions, such as southern Yunnan, China, and Myanmar, Laos, Indonesia [8]. Besides *A. konjac* and *A. albus*, it has the largest planting area in China. It has the characteristics of strong disease resistance, high yield and high KGM content [9, 10], and has good production potential and economic benefit [11–13]. In addition to the main KGM, konjac corms also contain starch and soluble sugar, protein, crude fiber, amino acids and fat [14]. Previous studies on the nutritional components of *A. bulbifer*, *A. konjac* and *A. albus* showed that the content of KGM was rich, the fat content was low, and the calorie content was low; the content of KGM was correlated with viscosity; the content of protein and amino acid decreased with the increase of cultivation years, while the content of crude polysaccharide increased first and then decreased; there was no obvious rule for trace elements [15–18]. But there is no systematic study on the nutritional components of *A. bulbifer*, the evaluation index is relatively single, and the quality research and comprehensive evaluation are relatively weak.

Correlation analysis, principal component analysis (PCA) and metabolome analysis are widely used in multi-index quality analysis of crops and comprehensive variety evaluation. Correlation analysis and PCA were applied to analyze the physiological characteristics and nutritional quality of maize Zhengdan 958 (Zd958) and Xianyu 335 (XY335) during the post-ripening process, and three principal components were extracted: yield factor, storage intolerance factor and digestion characteristic factor, according to the F value of comprehensive evaluation, reference basis could be provided for the post-ripening quality and post-ripening time of new maize [19]. By correlation analysis, PCA and variability, a comprehensive evaluation model was constructed for 14 kinds of *Malus* fruits, and 4 kinds of fruits with better quality were screened out, cluster analysis and sensory evaluation further verified the reliability of the model [20]. The contents of 6 tea varieties were determined, and the correlation analysis showed that the biosynthesis of metabolites was highly correlated, PCA could clearly distinguish spring tea from summer tea [21]. Metabolomics was used to detect the main substance composition of 5 different plum varieties, and the key substances which might lead to the difference between late ripening Sanhua plum and Dami plum were screened out by differential metabolite analysis [22].

In this study, we assumed that the quality of different varieties was different, and KGM was correlated with quality indicators such as viscosity and starch. Given the rich germplasm resources of *A. bulbifer*, we selected five isolated and identified cultivars that significant phenotypic variations with stable heritability (‘YunRe1701’, ‘YunRe1707’, ‘YunRe1709’, ‘YunRe1710’, and ‘YunRe1726’) as experimental materials to analyze the growth, yield, quality and metabolome. The correlation analysis was used to reveal the correlation among the quality indexes, to study the main nutrient composition of *A. bulbifer*. PCA was used to evaluate the quality of 5 different varieties, which provided reference for nutrient composition, quality evaluation and development and utilization of excellent varieties.

## Materials and methods

### Plant materials and growth conditions

The germplasm resources ‘YunRe1701’, ‘YunRe1707’, ‘YunRe1709’, ‘YunRe1710’ and ‘YunRe1726’ were developed as follows: wild *A. bulbifer* germplasm collected by the Yunnan Institute of Tropical Crops in 2016 was domesticated and cultivated from 2017 to 2020. Phenotypically specific and stably heritable accessions were screened and isolated to establish these varieties. Identified as *A. bulbifer* by Associate Researcher Yanxiong Gong of the same institute, these materials are preserved in its Konjac Germplasm Resource Nursery and were used as experimental subjects in this study.

In order to ensure the accuracy of the experiment and reduce the error of the experiment, healthy corms with no diseases and insect pests and 100 - 150 g per unit were selected as the propagation materials and planted in April 2022 and 2023 (There was no significant difference in the two-year data of each cultivar, *p* < 0.05, Table 1). A randomized block design was adopted, with 3 repeated plots for each variety, each plot area of 15 m^2^, 30 plants per plot, and standardized field management. The experimental site (22°00′24″N, 100°46′14″E) has a shade degree of 75%, an altitude of 580 m, an annual average temperature of 23.5℃, an annual rainfall of 1214.9 mm, and an annual sunshine duration of 2126.6 h. It belongs to a tropical monsoon climate with distinct dry and wet seasons.

**Table 1:**
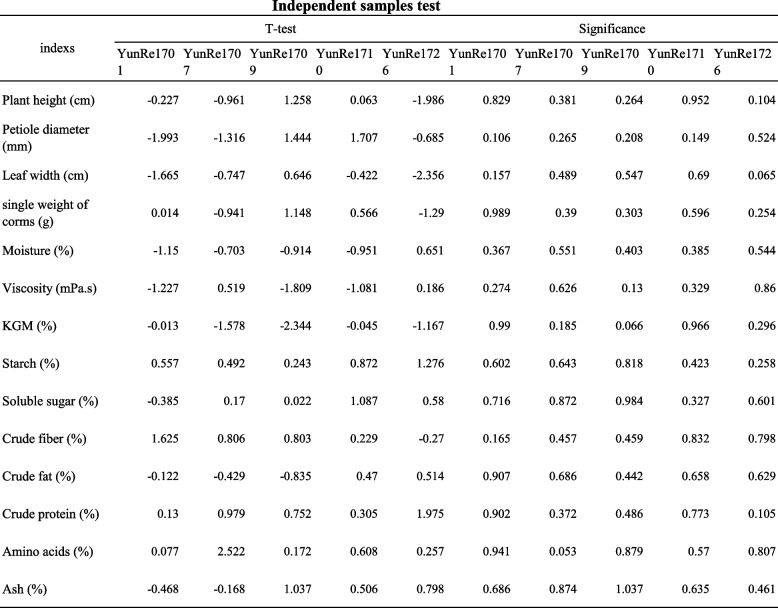
T-test analysis of samples from different years

### Plant growth and yield determination

In early August, when the plant height, leaf width and petiole diameter of each variety tended to be stable, 30 plants were selected from each plot, and the petiole diameter of each plant close to the ground was measured with a vernier caliper; the plant height was measured with a tape measure from the petiole base close to the ground to the petiole bifurcation; the leaf width was the average value of the length and width of the whole leaf measured with the tape measure. At the end of November, after the degradation of konjac plants, 30 corms were extracted from each plot, the surface soil was removed, and the corms were weighed on an electronic scale (precision 0.01 g) to calculate the single weight of each variety (line).

### Corm moisture content

After corms are tested, at least 3 biological duplicate corms are selected from each plot and returned to the room for cleaning. After peeled, the fresh weight is weighed, then sliced and placed in an oven at 105℃ for 30 min, then adjusted to 60℃ and dried at constant temperature, and the dry weight is recorded when the dry weight of the dry piece is constant. Preparation of konjac powder after drying: put the dried slices in a pulverizer and grind them into powder, and then store them after passing through a 40-mesh sieve for quality index determination.

### KGM content

#### Preparation of standard curve

Weigh 0.1000 g dried glucose (analytical grade), dissolve in distilled water and dilute to 100 mL in a volumetric flask. Aliquot 0.0, 0.4, 0.8, 1.2, 1.6, and 2.0 mL into six 25 mL volumetric flasks, adjust to 2 mL with distilled water. Add 1.5 mL 3,5-dinitrosalicylic acid (DNS) reagent to each, heat in 100 °C water bath for 5 min, cool and dilute to the mark. Using the 0.0 mL sample as blank, measure absorbance at 550 nm. A standard calibration curve was plotted with glucose mass (mg) as the X-axis and absorbance as the Y-axis.

#### Determination of glucomannan content

Preparation of glucomannan extract: 0.2 g konjac powder and add 50 mL NaOH-formic acid buffer (stir 2–3 h, stand overnight) into a 100 mL volumetric flask.. Dilute to the mark with buffer, centrifuge (4000 rpm, 20 min), collect supernatant.

Preparation of glucomannan hydrolysate: transfer 5 mL of the extract to a 25 mL volumetric flask. Add 2.5 mL H_2_SO_4_ (3 mol/L), hydrolyze (100 °C, 1.5 h, sealed). Cool, neutralize with 2.5 mL NaOH (6 mol/L), mix, and constant volume to scale.

Measurement: add 2 mL of the extract, hydrolysate, or distilled water (as control) to three separate 25 mL volumetric flasks. Add 1.5 mL of DNS reagent to each, heat in a 100 °C water bath for 5 min, cool, and dilute to volume with distilled water. Zero the spectrophotometer using a mixture of 2.0 mL distilled water and 1.5 mL DNS reagent. Measure absorbance at 550 nm [23].

### Viscosity measurement

Weigh 8.00 g sample and add it into a beaker containing 500 mL deionized water. After stirring evenly, place it in a constant temperature water bath at 30 ± 1℃, stir with an electric stirrer at a rotation speed of 200 r/min. After 1 h, measure the viscosity for the first time (NDJ- 5S, Shanghai Youyi); then repeat the measurement every 0.5 h until the reading reaches the maximum value and obviously begins to decrease. Record the maximum value as viscosity (mPa·s).

### Starch

Sample preparation: weigh 2–5 g konjac powder, sequentially degrease and remove sugars with 50 mL ether and 100 mL 85% ethanol. Transfer the residue to a 250 mL beaker, add 50 mL water, boil in a water bath for 15 min, cool to 60 °C, add 20 mL amylase solution, stir for 1 h, and test with iodine until no color develops. Dilute to 250 mL, filter (discarding the initial filtrate). Transfer 50 mL filtrate, add 5 mL HCl (1:1), reflux with a condenser for 1 h, cool, neutralize to neutrality, and dilute to 100 mL.

Calibration: add 5 mL each of alkaline tartrate copper Solution A and B, 10 mL water, glass beads, and 9 mL glucose standard (1 mg/mL) to a 150 mL conical flask. Heat to boiling, titrate with glucose standard until blue color disappears, record volume, and calculate m₁ (glucose mass in mg per 10 mL copper solution).

Sample assay: replace glucose standard with sample solution, titrate following the same procedure, and record volume consumed.

Blank determination: add 20 mL water and 20 mL amylase solution, titrate the blank, calculate volume discrepancy, and convert to glucose mass.

Calculation: starch content is calculated using C.L. Zhu’s method [24].

### Soluble sugar

Take 0.25 g konjac powder and 25 mL acetic acid solution (5%) into a 50 mL triangle bottle, plug, and oscillate for 30 min (rotation speed > 150r/min); The filtrate is filtered, retained, and determined using a continuous flow analyzer [25].

### Fiber

Weigh 5 g of sample and boil 200 mL of sulfuric acid solution (1.25%) in a 500 mL Erlenmeyer flask, heated to a slightly boiling state, and kept for 30 min, shaking once every 5 min. After that, filter with linen cloth and wash with boiling water until the washing solution is not acidic. Wash the residue on linen cloth into the original Erlenmeyer flask with 200 mL of boiling 1.25% potassium hydroxide solution, repeat the above steps 2–3 times, suction and drain, and bake the cyanosis and contents in an oven at 105℃ to constant weight [26].

$$\text{Fiber}\;\text{content}\;(\%)\;=\;\text G\;/\;\text m\;\times\;100\%$$

where G and m represent the residual mass of the sample (g) and the mass of the sample (g).

### Crude fat

Weigh 5 g (m_1_) of konjac flour into an extraction thimble, and place some carborundum in a pre-dried flask (m_2)_. Assemble the Soxhlet extractor with the flask. Position the thimble in the extractor and perform petroleum ether extraction for 6 hours. Distill off the solvent until minimal residue remains in the flask. Add 2 mL of acetone to the flask, swirl the flask, and gently warm it on a heating apparatus to evaporate the acetone. Remove trace acetone by air blowing. Dry the residue in a 103 °C oven for 10 min, cool in a desiccator, and record the final mass (m_3_) [27].

$$\text{Crude}\;\text {fat}\;(\%)\;=\;({\text m}_3-{\text m}_2)/{\text m}_1\;\times\;100\%$$

### Crude protein

Weigh about 0.5 g konjac flour into a 100 mL conical flask, add 25 mL of 0.5% acetic acid solution, heat to boiling for 15 min, and then suction filter. Transfer filter paper and precipitate to a digestion tube, add 0.1 g mercuric oxide, 1 g potassium sulfate and 5 mL concentrated sulfuric acid for digestion for 1 h (150℃), then digest at 370℃ for 4 h, cool and dilute to volume with water; and 5 ammonium sulfate solutions with different concentrations were established as standard curves [28].

### Amino acids

Sample preparation: weigh 3.000 g konjac flour into a 500 mL conical flask. Add 450 mL deionized water, extract in a 100 °C water bath for 45 min, filter under vacuum, and transfer filtrate to a 500 mL volumetric flask. Dilute to volume with water [29].

Sample analysis: transfer 1 mL sample solution to a 25 mL colorimetric tube. Add 0.5 mL phosphate buffer (pH 8.0) and 2% ninhydrin solution. Heat in boiling water bath for 15 min, dilute to volume, and let stand for 10 min. Measure absorbance at 570 nm using reagent blank as reference.

Standard curve: aliquot 0.0, 1.0, 1.5, 2.0, 2.5, and 3.0 mL of glutamic acid standard solution (10 mg/mL) into six 25 mL colorimetric tubes. Repeat sample procedure and measure absorbance. Plot glutamic acid concentration vs. absorbance.

Calculation:

$$\text{Amino}\;\text{acids}\;(\%)\;=\;(\text C/1000\times{\text V}_1/{\text V}_2)/(\text m\times{\omega})\times100\%$$

Where: C: glutamic acid concentration from standard curve (mg), V_1_: total sample volume (mL), V_2_: aliquot volume (mL), m: sample mass (g), ω: sample water content (%).

### Ash

Put the empty crucible with half open lid in muffle furnace at 550℃ and burn it to constant weight (mass difference between two times is less than 0.0005 g). Weigh 2 - 3 g konjac powder in porcelain crucible with known mass and record the mass of both (0.0001 g). Heat it with low heat until the sample smokes. After the smoke is over, continue burning for 15 min. Move the crucible into muffle furnace and heat it to 550℃ and burn it to constant weight [30].

$$\text{Ash}\;(\%)=\;({\text m}_1-{\text m}_2)/({\text m}_3-\;{\text m}_2)\times100\%$$

where m_1_ represents the mass (g) of the crucible and ash, m_2_ represents the mass (g) of the empty crucible, and m_3_ represents the mass (g) of the crucible and sample.

### Metabonomic detection

The freeze-dried corm was crushed using a mixer mill (MM 400, Retsch) with a zirconia bead for 1.5 min at 30 Hz, with three biological replicates per variety. 100 mg powder was weighted and extracted overnight at 4℃ with 1.0 ml 70% aqueous methanol. Following centrifugation at 10,000 × g for 10 min, the extracts were absorbed (CNWBONDCarbon-GCB SPE Cartridge, 250 mg, 3 mL; ANPEL, Shanghai,China, www.anpel.com.cn/cnw) and filtrated (SCAA- 104, 0.22 μm pore size; ANPEL,Shanghai, China, http://www.anpel.com.cn/) before liquid chromatograph mass spectrometer (LC-MS) analysis.

The sample extracts were analyzed using an liquid chromatography electrospray ionization tandem mass spectrometry (LC-ESI-MS/MS) system (HPLC, Shim-pack UFLC SHIMADZU CBM30 A system, www.shimadzu.com.cn/; MS, Applied Biosystems 6500 Q TRAP, www.appliedbiosystems.com.cn/). The analytical conditions were as follows, HPLC: column, Waters ACQUITY UPLC HSS T3 C18 (1.8 µm, 2.1 mm* 100 mm); solvent system, water (0.04% acetic acid): acetonitrile (0.04% acetic acid); gradient program, 95:5 V/V at 0 min, 5:95 V/V at 11 min, 5:95 V/V at 12 min, 95:5 V/V at 12.1 min, 95:5 V/V at 15 min; flow rate, 0.40 mL/min; temperature, 40 °C; injection volume: 2 μL. The effluent was alternatively connected to an ESI-triple quadrupole-linear ion trap (Q TRAP)-MS.

Linear ion trap (LIT) and triple quadrupole (QQQ) scans were acquired on a triple quadrupole-linear ion trap mass spectrometer (Q TRAP), API 6500 Q TRAP LC/MS/MS System, equipped with an ESI Turbo Ion-Spray interface, operating in a positive ion mode and controlled by Analyst 1.6.3 software (AB Sciex). The ESI source operation parameters were as follows: ion source, turbo spray; source temperature 500 °C; ion spray voltage (IS) 5500 V; ion source gas I (GSI), gas II (GSII), curtain gas (CUR) were set at 55, 60, and 25.0 psi, respectively; the collision gas (CAD) was high. Instrument tuning and mass calibration were performed with 10 and 100 μmol/L polypropylene glycol solutions in QQQ and LIT modes, respectively. QQQ scans were acquired as multiple reaction monitoring (MRM) experiments with collision gas (nitrogen) set to 5 psi. Declustering potential (DP) and collision energy (CE) for individual MRM transitions was done with further DP and CE optimization. A specific set of MRM transitions were monitored for each period according to the metabolites eluted within this period [31].

### Statistical analysis

The data were processed by analysis of variance (ANOVA) using SPSS software (version 26), with a significance level of 0.05, and means were separated by Duncan’s multiple range test. Results are expressed as mean ± standard error (M±SE). Bioinformatics cloud tools (https://www.omicsshare.com/tools/) were utilized for KEGG annotation, enrichment analysis, and differentially accumulated metabolites (DAMs) analysis of the metabolic profile data. Origin 2021 was employed for bar chart plotting, PCA and correlation analysis, TBtools for heatmap visualization of differential metabolites, and Adobe Illustrator 2022 for artwork refinement.

## Results

### Analysis on growth and yield of different konjac varieties

**Fig 1:**
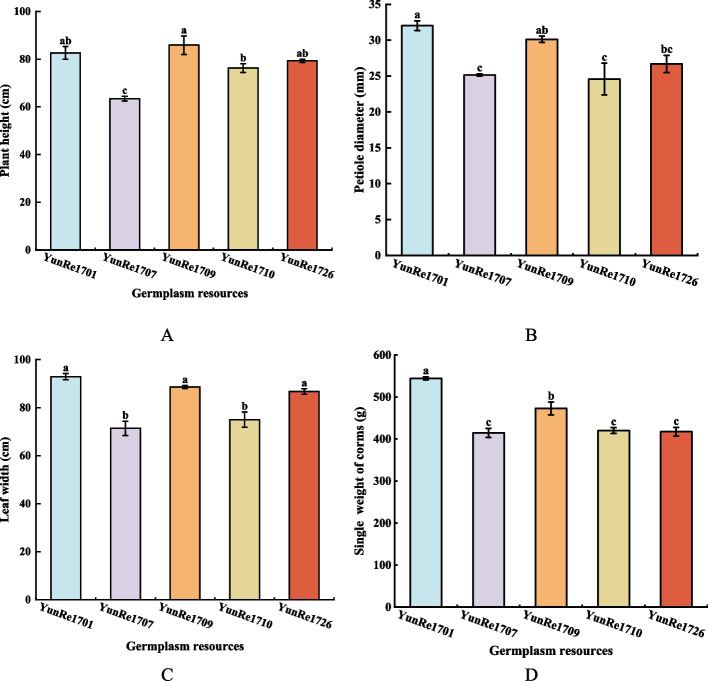
Comparison of external quality among different konjac germplasm resources. **A**: plant height; **B**: petiole diameter; **C**: leaf width; **D**: single weight of corms.

The petiole diameter, plant height, leaf width and yield of 5 varieties of *A. bulbifer* were as shown in Figure 1. ‘YunRe1701’ and ‘YunRe1709’ showed vigorous growth, ‘YunRe1726’ followed, and the trend of corm weight was the same. Among the 5 *A. bulbifer*, the plants of ‘YunRe1701’, ‘YunRe1709’ and ‘YunRe1726’ were higher, followed by ‘YunRe1710’. ‘YunRe1707’ demonstrated the shortest stature, with reductions of 23.21%, 26.11%, 20.00%, and 16.78% compared to ‘YunRe1701’, ‘YunRe1709’, ‘YunRe1726’, and ‘YunRe1710’, respectively (Figure 1A). The petiole diameters of ‘YunRe1701’ and ‘YunRe1709’ were thicker, with diameters of 32.0 mm and 30.11 mm, respectively. ‘YunRe1726’ followed by 26.69 mm; ‘YunRe1707’ and ‘YunRe1710’ are the smallest at 25.14 mm and 24.58 mm, significantly lower than ‘YunRe1701’ and ‘YunRe1709’ (Figure 1B). The leaf width of ‘YunRe1701’, ‘YunRe1709’ and ‘YunRe1726’ were 92.90 cm, 88.63 cm and 86.68 cm respectively, which were significantly higher than those of ‘YunRe1707’ and ‘YunRe1710’ (Figure 1C). After harvesting konjac in the plot, the corm weight was measured. Among the 5 varieties, ‘YunRe1701’ had the highest bulbous unit weight (544.47 g), which was significantly higher than other varieties; ‘YunRe1709’ was the second (472.89 g); ‘YunRe1710’, ‘YunRe1726’ and ‘YunRe1707’ followed, and there was no significant difference among them (Figure 1D). Based on the above analysis, ‘YunRe1701’, ‘YunRe1709’ and ‘YunRe1726’ were larger plants with higher petiole diameter, plant height and leaf width, while ‘YunRe1707’ and ‘YunRe1710’ were smaller plants with smaller petiole diameter, plant height and leaf width.

### Correlation analysis between growth and yield

**Fig 2:**
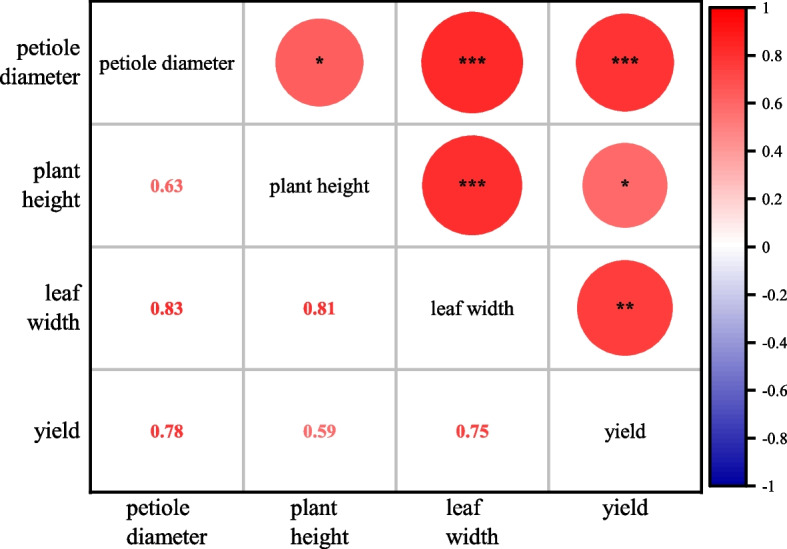
Correlation analysis of konjac growth and yield. “*” indicates significant difference at the 0.05 level, “**” indicates highly significant difference at the 0.01 level, “***” indicates highly significant difference at the 0.001 level, the same below

There is an interactive relationship between aboveground and underground growth of plants, which is of great significance to improve yield, plant shape cultivation and germplasm selection. In this study, correlation analysis was carried out on petiole diameter, plant height, leaf width and yield of 5 konjac germplasm resources, and the results were shown in Figure 2. The yield was significantly correlated with petiole diameter (*r* = 0.78, *p* < 0.001), leaf width (*r* = 0.75, *p* < 0.01), and plant height (*r* = 0.59, *p* < 0.05). There was a significant positive correlation between petiole diameter and leaf width, with a correlation coefficient of 0.83. The correlation coefficient between plant height and leaf width was 0.81. Leaf width was significantly correlated with petiole diameter, plant height and yield. To sum up, there was significant positive correlation among petiole diameter, plant height, leaf width and yield, and the correlation coefficient was 0.59–0.83.

### Quality analysis of konjac corm

#### Physical quality analysis

**Fig 3:**
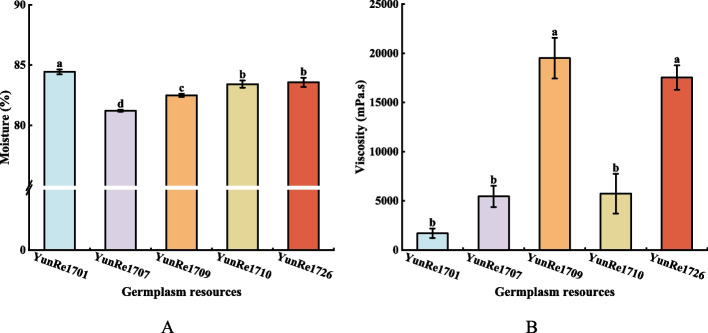
Comparison of moisture (**A**) and viscosity (**B**) among different konjac germplasm resources.

Water is an important component in the organism and plays an important role in metabolism and substance composition. The moisture of corms of 5 *A. bulbifer* varied from 81.20% to 84.44%. Among them, ‘YunRe1701’ had the highest moisture, which was significantly higher than that of other varieties, followed by ‘YunRe1726’, ‘YunRe1710’, ‘YunRe1709’ and ‘YunRe1707’ (Figure 3A). The moisture of corms of 5 *A. bulbifer* varied significantly, indicating that there were significant differences in corm quality.

Konjac powder has strong viscosity after being dissolved in water, which plays an important role in its utilization and can be used in papermaking, thickener, degradable film and so on. The viscosity of the five varieties was different, ‘YunRe1709’ and ‘YunRe1726’ demonstrated significantly higher viscosities compared to the other three varieties, followed by ‘YunRe1710’, ‘YunRe1707’, and ‘YunRe1701’ in descending order. Notably, the aqueous solution of ‘YunRe1701’ displayed lower viscosity and failed to form a cohesive viscous hydrosol (Figure 3B).

#### Active ingredient analysis

**Fig 4:**
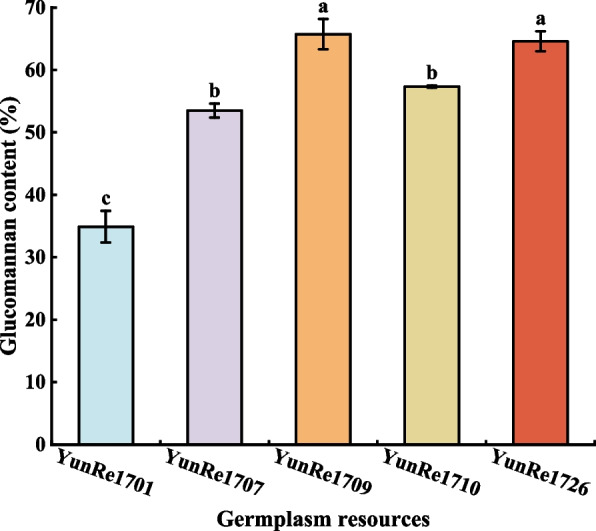
Comparison of KGM content among different konjac

KGM is the most important active component in konjac corm, which plays an important role in treating diabetes, obesity and reducing blood glucose and lipids. It can also be used in industry, medicine, food and other industries by its film-forming property, thickening property, gelation property and water solubility. It is an important index to evaluate the quality of konjac.The KGM content of 5 konjac germplasm resources was shown in Figure 4. ‘YunRe1709’ and ‘YunRe1726’ had the highest KGM content, which was significantly higher than the other three varieties, and their konjac powder reached the first-class konjac powder standard. The content of ‘YunRe1707’ and ‘YunRe1710’ KGM were the second, reaching the grade 3 standard of ordinary konjac powder [22]. ‘YunRe1701’ had the lowest content, accounting for only 53% to 65% of the other 4 varieties.

#### Nutrient analysis

**Fig 5:**
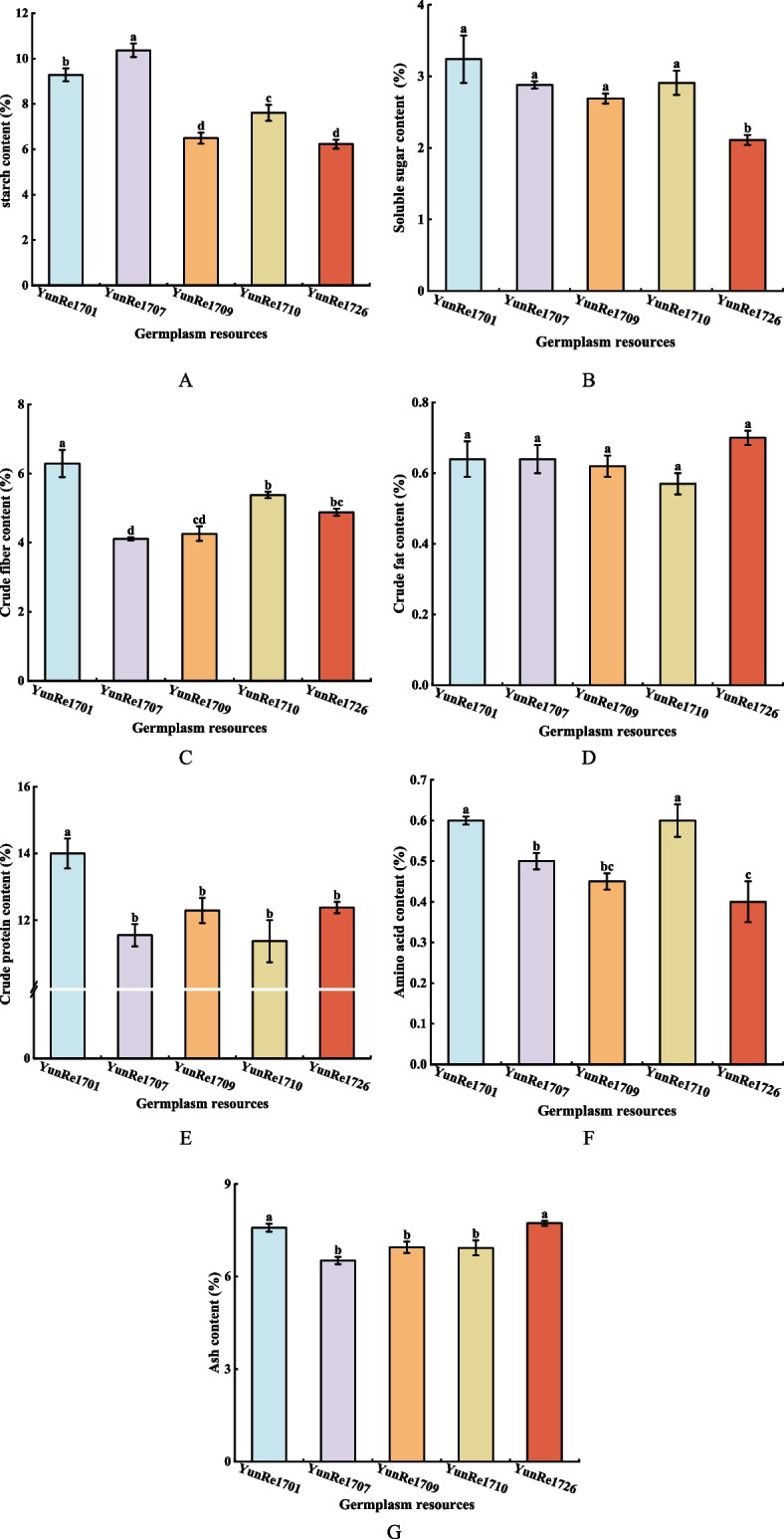
Nutritional components of konjac. **A**: starch; **B**: soluble sugar; **C**: crude fiber; **D**: crude fat; **E**: crude protein; **F**: amino acid; **G**: ash.

The starch content of 5 konjac germplasm resources ranged from 6.23% to 10.36% (Figure 5A). ‘YunRe1707’ was significantly higher than other varieties. ‘YunRe1701’ and ‘YunRe1710’ were the second, and there were significant differences between them. The starch content of ‘YunRe1709’ and ‘YunRe1726’ was the lowest, which was obviously lower than that of other varieties. The results indicated that there were significant differences in starch content among 5 germplasm resources.

In addition to KGM, starch and other polysaccharides, konjac corms also contain a small part of sucrose, fructose, glucose and other soluble sugars, which can be used as a medium for energy storage or transfer. The soluble sugar content of 5 konjac germplasm resources ranged from 2.11% to 3.24% (Figure 5B). The content of ‘YunRe1726’ was significantly lower than that of other varieties, followed by ‘YunRe1709’, and the overall performance was ‘YunRe1726’ < ‘YunRe1709’ < ‘YunRe1707’ < ‘YunRe1710’ < ‘YunRe1701’.

The crude fiber in konjac can promote intestinal peristalsis, reduce the absorption of nutrients and some small molecules in the intestine, and thus play an important role in relieving constipation, treating obesity, diabetes and reducing blood sugar and lipids. The crude fiber content of 5 konjac germplasm resources ranged from 4.11% to 6.29% (Figure 5C), and ‘YunRe1701’ had the highest content, which was significantly higher than other varieties. ‘YunRe1710’ ranked second, significantly higher than ‘YunRe1707’ and ‘YunRe1709’; the overall performance was ‘YunRe1701’ > ‘YunRe1710’ > ‘YunRe1726’ > ‘YunRe1709’ > ‘YunRe1707’.

Fat is an important form of energy storage for animals and plants, which plays an important role in maintaining normal temperature and protecting healthy heart, blood vessels and liver, but excessive intake can also lead to obesity, blood sugar and blood pressure. The crude fat content of 5 konjac germplasm resources was 0.57%− 0.70%, ‘YunRe1726’ > ‘YunRe1701’/‘YunRe1707’ > ‘YunRe1709’ > ‘YunRe1710’, and there was no significant difference among the 5 konjac germplasm resources (Figure 5D). Based on the above analysis, the results showed that the fat content of konjac was low, and there was no significant difference between varieties, which further provided scientific basis for its use as health food with low energy and low calories.

Crude protein is an important nutrient component in plant body, and also an important index to measure its nutritional quality and economic value. The crude protein content of 5 konjac germplasm resources ranged from 11.37% to 14.00% (Figure 5E), and ‘YunRe1701’ had the highest content, which was significantly higher than other varieties. There was no significant difference between ‘YunRe1707’, ‘YunRe1709’, ‘YunRe1710’ and ‘YunRe1726’. Based on the above analysis, ‘YunRe1701’ exhibits high levels of starch, soluble sugars, crude fiber, crude fat, and crude protein, indicating substantial recycling value in its processing byproducts.

Amino acids play an important role in glycolysis, hormone level regulation, energy supply and nutrient regulation in the body. The amino acid content of 5 konjac varieties ranged from 0.40% to 0.60%, among which ‘YunRe1701’ and ‘YunRe1710’ had higher amino acid content than other varieties. The content of ‘YunRe1726’ was the lowest, significantly lower than that of ‘YunRe1701’, ‘YunRe1707’ and ‘YunRe1710’ (Figure 5F).

The ash content of 5 konjac germplasm resources after high temperature burning is shown in Figure 5G. ‘YunRe1701’ and ‘YunRe1726’ had higher ash content, which was significantly higher than that of the other 3 varieties, and the other 3 had no significant difference. To sum up, the ash content of konjac corm is higher, indicating that it contains more mineral nutrients such as calcium, magnesium, potassium and iron, which is conducive to the development of its utilization value.

#### Quality correlation analysis

**Fig 6:**
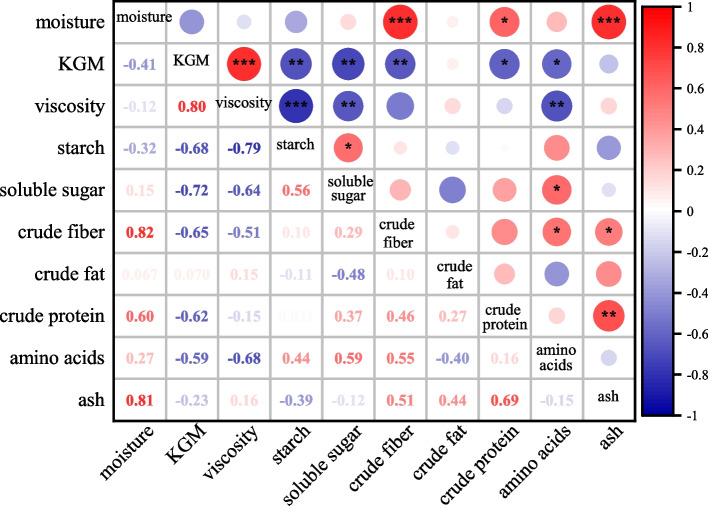
Correlation analysis of konjac quality

The correlation among KGM, viscosity, starch and other quality indexes of *A. bulbifer* is shown in Figure 6. The moisture of fresh corm was positively correlated with crude fiber and ash at *p* < 0.001, and with crude protein at *p* < 0.05. KGM was positively correlated with viscosity at *p* < 0.001 (*r* = 0.80); there was significant negative correlation with starch, soluble sugar and crude fiber at *p* < 0.01, and the correlation coefficient was 0.65–0.72; there was significant negative correlation with crude protein and amino acid. The correlation of viscosity was similar to the KGM trend. Starch was positively correlated with soluble sugar (*r* = 0.56), and both were negatively correlated with KGM and viscosity. Crude fiber had significant positive correlation with moisture(*r* = 0.82), amino acid (*r* = 0.55) and ash (*r* = 0.51), and significant negative correlation with KGM (*r* = 0.65). There was no correlation between crude fat and KGM. Crude protein was positively correlated with ash (*r* = 0.69) and moisture (*r* = 0.60), and negatively correlated with KGM (*r* = 0.62). Amino acids were positively correlated with soluble sugar (*r* = 0.59) and crude fiber (*r* = 0.55), negatively correlated with viscosity (*r* = 0.68) and KGM (*r* = 0.59).

### Principal component analysis (PCA)

**Fig 7:**
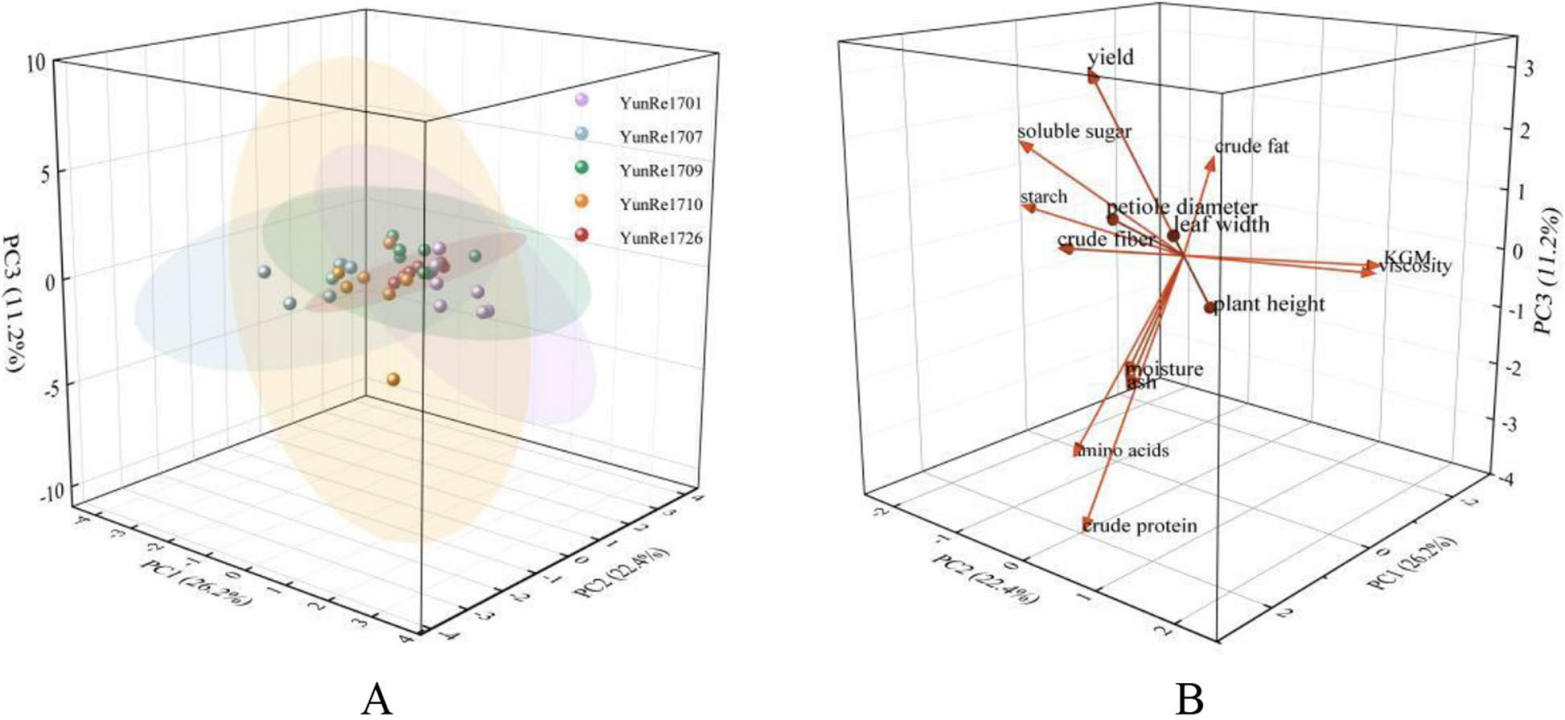
Principal component analysis (PCA) score plot (**A**) and loading plot (**B**) of 5 konjac varieties.

In order to further understand the relationship between varieties of *A. bulbifer*, we used biochemical data such as growth, yield and quality as input variables for principal component analysis, the principal components were screened based on the criterion of eigenvalues > 1, and the results are shown in Figure 7. The first three principal components explained 59.78% of the observed variation, with PC1 contributing 26.18%, PC2 contributing 22.40%, and PC3 contributing 11.19%. PC1 showed positive correlations with petiole diameter, plant height, leaf width, yield, and crude protein, ash, moisture, as well as crude fiber, soluble sugar and starch; but it exhibited negative correlations with KGM and viscosity. Conversely, PC2 displayed positive correlations with KGM and viscosity, as well as plant height and leaf width; but it showed negative correlations with starch, soluble sugar, crude fiber and amino acids. PC3 positively correlated with yield, crude fat, and soluble sugar, and showed a negative correlation with crude protein and amino acids. ‘YunRe1701’ consistently scored positively along PC1, the corm contains rich crude fiber and crude protein, and the processing waste has high recycling value. While ‘YunRe1709’ and ‘YunRe1726’ consistently scored positively along PC2, and exhibited strong correlations with KGM contents, the KGM content of corms is high, viscosity is strong, and has good nutritional value and economic benefit (Figure 7).

### Metabolome analysis

**Fig 8:**
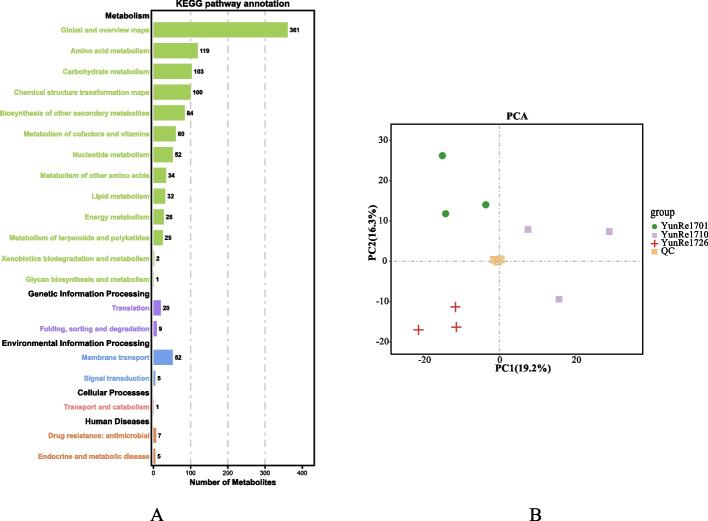
KEGG annotation (**A**) and quality control PCA analysis (**B**) of the corm metabolite of 3 konjac varieties.

In order to fully understand the metabolic composition of *A. bulbifer*, we used ultra performance liquid chromatography (UPLC) and Tandem mass spectrometry (MS/MS) to analyze the metabolomics of ‘YunRe1701’, ‘YunRe1710’ and ‘YunRe1726’ corms, and 945 metabolites were detected. Among them, 575 primary metabolites were identified, including 187 amino acids and derivatives, 5 alcohols and polyols, 70 nucleotides and its derivatives, 98 carbohydrates and its derivatives, 21 vitamins, 89 organic acids and its derivatives, and 105 lipids. 370 secondary metabolites were identified, including 26 amines, 23 phenylpropanoids and polyketides, 4 benzene and substituted derivatives, 3 polyamines, 29 phenols and its derivatives, 22 phenolic acids, 118 flavonoids, 27 alkaloids and derivatives, 42 terpenoids, 2 organic sulfides, 3 organosulfur compounds, 57 organoheterocyclic compounds and 14 phytohormones. KEGG annotation of the above metabolites indicated that the compounds in the bulb were mainly involved in the global and overview maps, amino acid metabolism, carbohydrate metabolism, chemical structure transformation maps, biosynthesis of other secondary metabolites, metabolism of cofactors and vitamins, nucleotide metabolism, metabolism of other amino acid metabolism, lipid metabolism, energy metabolism, metabolism of terpenoid and polyketone, and other metabolic pathways, as well as genetic information processing, environmental information processing and other processes (Figure 8A). In order to preliminarily understand the overall metabolic differences and the degree of differences among the *A. bulbifer* varieties, we analyzed the samples of ‘YunRe1701’, ‘YunRe1710’ and ‘YunRe1726’, as well as the quality control samples. In the PCA (Figure 8B), QC samples were densely distributed, and the 3 varieties were clearly distinguished along PC1 and PC2, which was consistent with the results of PCA analysis based on growth, yield and quality. The results showed that the data were reliable and the metabolites of different varieties were different, which laid a foundation for the subsequent analysis of the metabolites of *A. bulbifer*.

### DAM screening and KEGG enrichment

**Fig 9:**
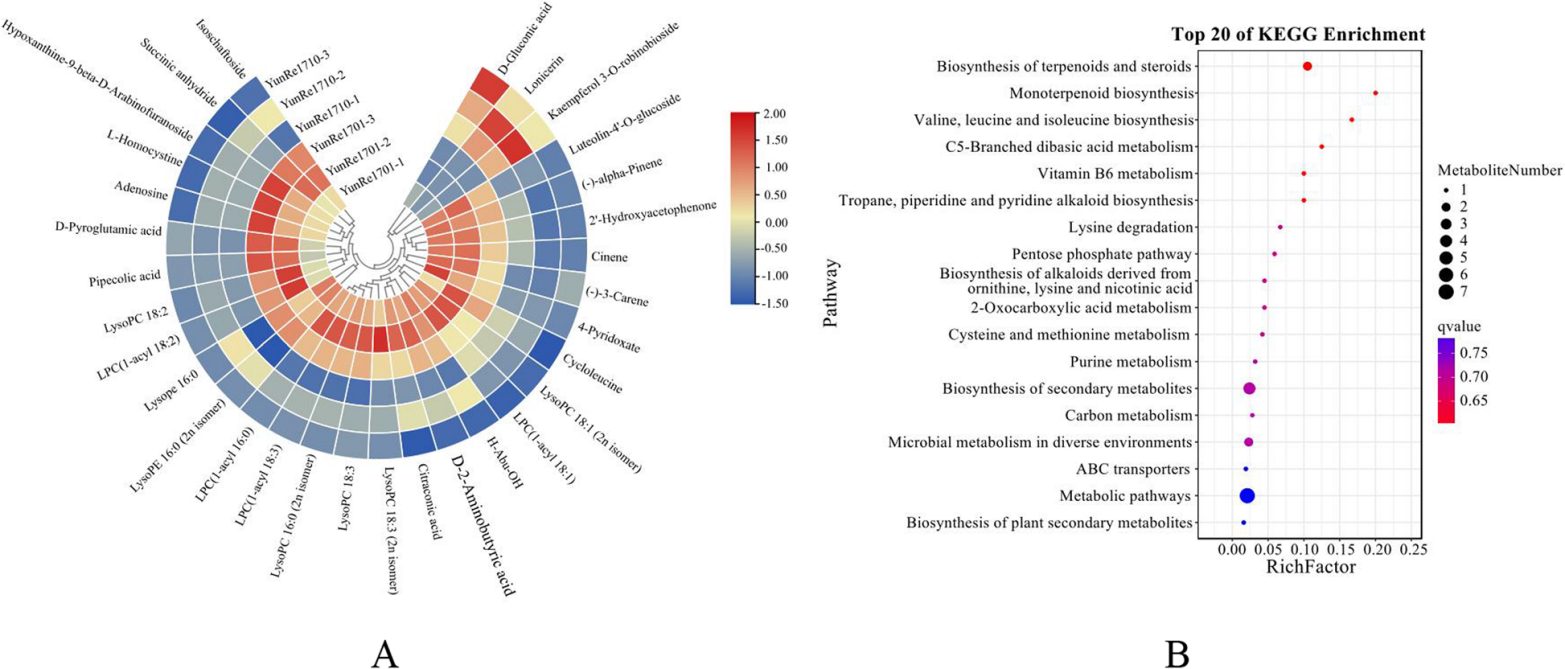
DAMs (**A**) and KEGG enrichment (**B**) of ‘YunRe1710’ VS ‘YunRe1701’.

In order to screen important differentially accumulated metabolites in *A. bulbifer* corms, we used variable projection importance (VIP) and fold change (Fold Change) were employed as the criteria for screening the DAMs. Metabolites meeting the conditions of a Fold Change ≥ 2 and a Fold Change ≤ 0.5, along with a VIP ≥ 1, were selected. Compared with ‘YunRe1701’, ‘YunRe1710’ had 31 DAMs, including 5 amino acids and derivatives, 1 phenol and its derivatives, 2 nucleotides and its derivatives, 4 flavonoids, 1 carbohydrates and its derivatives, 3 terpenoids, 2 organic acids and its derivatives, 1 organoheterocyclic compound and 12 lipids, 3 of which were up-regulated and 28 down-regulated (Figure 9A. KEGG showed that DAMs were mainly enriched in metabolic pathways and biosynthesis of secondary metabolites (Figure 9B).

**Fig 10:**
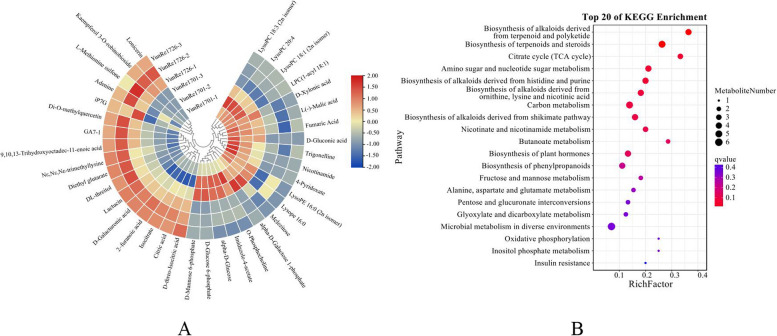
DAMs (**A**) and KEGG enrichment (**B**) of ‘YunRe1726’ VS ‘YunRe1701’

Compared with ‘YunRe1701’, ‘YunRe1726’ had 37 DAMs, including 2 amino acids and derivatives, 1 amine, 3 flavonoids, 1 alkaloid and its derivatives, 8 carbohydrates and its derivatives, 1 vitamin, 8 organic acids and its derivatives, 3 organoheterocyclic compounds, 8 lipids and 2 phytohormones. Among them, 17 DAMs were down-regulated and 20 were up-regulated (Figure 10A). KEGG enrichment analysis shows that DAMs were mainly involved in biosynthesis of alkaloids derived from terpenoid and polyketide, biosynthesis of terpenoids and steroids, citrate cycle (TCA cycle), amino sugar and nucleotide sugar metabolism, carbon metabolism, nicotinate and nicotinamide metabolism, butanoate metabolism, biosynthesis of plant hormones, biosynthesis of phenylpropanoids, fructose and mannose metabolism, and alanine, aspartate and glutamate metabolism, pentose and glucuronate interconversions, glyoxylate and dicarboxylate metabolism, microbial metabolism in diverse environments in different environments and so on (Figure 10B).

**Fig 11:**
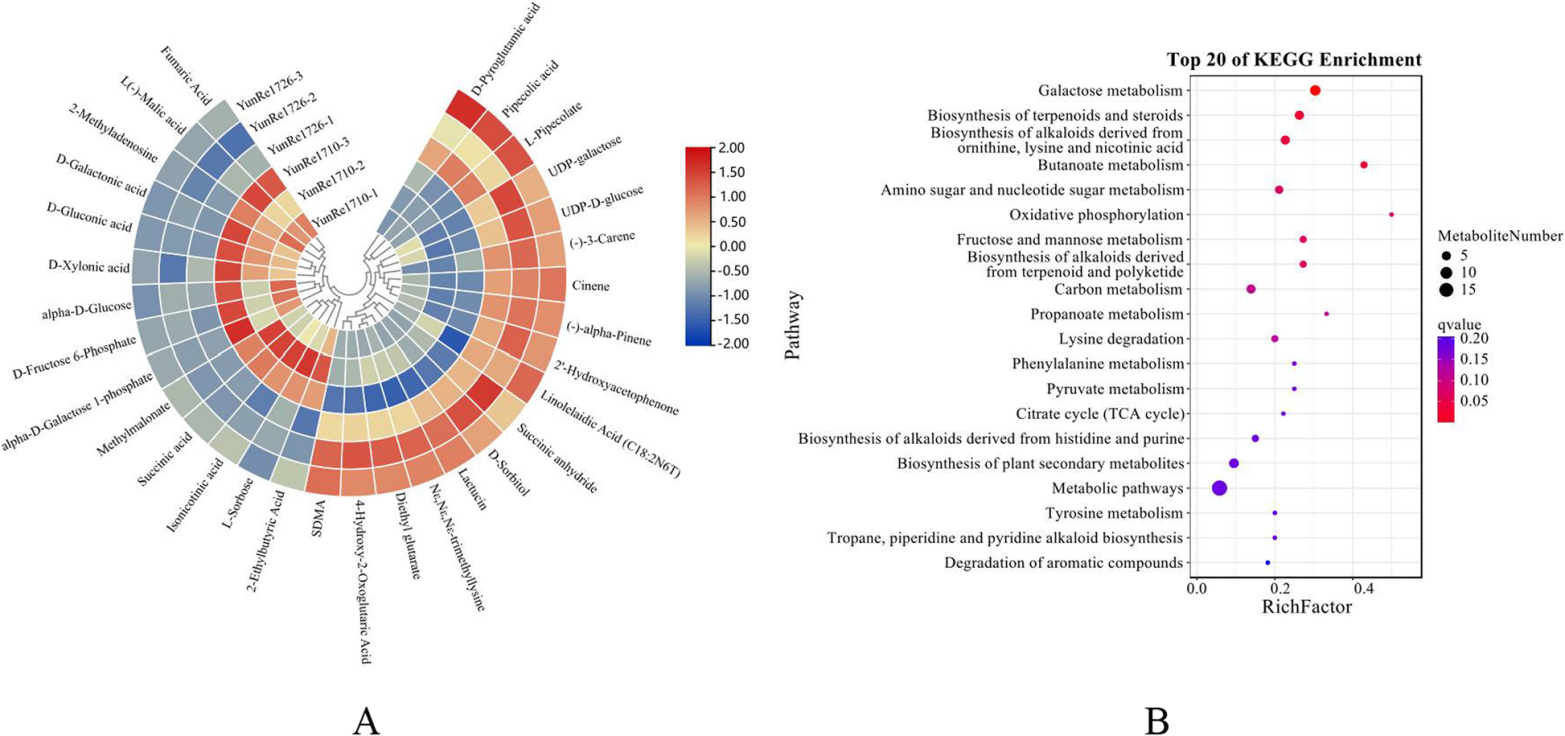
DAMs (**A**) and KEGG enrichment (**B**) of ‘YunRe1726’ VS ‘YunRe1710’.

Compared with ‘YunRe1710’, ‘YunRe1726’ had 31 DAMs, including 5 amino acids and their derivatives, 1 phenol and its derivatives, 3 nucleotides and its derivatives, 6 carbohydrates and its derivatives, 3 terpenes, 1 vitamin, 8 organic acids and its derivatives, 1 organoheterocyclic compound and 3 lipids, of which 17 DAMs were up-regulated, 14 were down-regulated(Figure 11A). KEGG enrichment analysis showed that DAMs were mainly involved in galactose metabolism, biosynthesis of terpenoids and steroids, biosynthesis of alkaloids derived from ornithine, lysine and nicotinic acid, butanoate metabolism, amino sugar and nucleotide sugar metabolism, fructose and mannose metabolism, carbon metabolism, lysine degradation, phenylalanine metabolism, metabolic pathways and biosynthesis of plant secondary metabolites (Figure 11B).

## Discussion

The aboveground and underground parts of the plant transport and supply nutrients, functioning as interdependent growth regulators that collectively ensure normal plant development [32, 33]. In konjac, the aboveground system comprises leaves and petioles, where leaves serve as the primary photosynthetic organs for energy acquisition. Organic matter synthesized through photosynthesis is transported to the underground corm through the petioles. Leaf dimensions correlate with photosynthetic efficiency, while plant height reflects nutritional status and growth vigor. Petiole diameter is associated with structural stability and lodging resistance. The underground part of konjac is mainly composed of abnormal stem (corm) and fibrous roots. Most of the organic matter synthesized by roots and leaves flows to corms, which is the main storage organ of KGM and other nutrients, consistent with the ‘source-sink’ theory in crop physiology. Correlation analyses revealed significant positive relationships among petiole diameter, plant height, leaf width, and corm yield (coefficients: 0.59–0.83). Yield exhibited highly significant correlations with petiole diameter (*r* = 0.78, *p* < 0.001) and leaf width (*r* = 0.75, *p* < 0.01), Leaf width was significantly positively correlated with the other three indexes (*r* = 0.75–0.83, *p* < 0.01), indicating that leaf width played an important role in the growth of konjac plants. Parallel patterns were observed in *Bletilla striata*, where yield had significant positive correlation with ground diameter, leaf width and leaf length (*p* < 0.01) [34]. Similarly, *Taxus chinensis* seedlings under 50% shade demonstrated enhanced aboveground biomass and root growth [35], and sweet potato treated with different biostimulants showed significantly increased stem diameter, tuber yield, and leaf area index [36]. These findings collectively validate the critical interplay between aboveground morphology and underground productivity in *A. bulbifer*, which is of great significance to the study of improving plant shape and yield by different light, fertilizer application, planting density and biological growth promoting bacteria.

In this study, 10 quality indexes (including KGM, starch and viscosity) were systematically analyzed. and the water content of fresh corms was about 80%, which was consistent with the results of Li L. and Li J.C. [14, 15]. KGM content ranged from 34.88% to 65.72%, with cultivars ‘YunRe1709’ and ‘YunRe1726’ exceeding 64%, demonstrating high yield, robust stress resistance, and promising commercial potential. A strong positive correlation was observed between KGM and viscosity (*r* = 0.8, *p* < 0.001), the main reason was that KGM had water swelling property, and the hydrosol formed had strong viscosity, which underpin its industrial applications. As the two primary components of konjac corms, KGM and starch significantly influence both functional utility and economic value. Notably, KGM displayed significant negative correlations with starch, soluble sugars, and crude fiber. Combined with previous research, the primary reason lies in the shared biosynthetic substrate of KGM, starch, soluble sugars, and crude fiber, which is sucrose converted from light energy absorbed by leaves. This sucrose is transformed into glucose- 1-phosphate through the actions of sucrose synthase, UDP-D-glucose pyrophosphorylase, hexokinase, and phosphoglucomutase. Subsequently, glucose- 1-phosphate generates starch under the catalysis of ADP-D-glucose pyrophosphorylase, starch synthase, and starch branching enzyme, while it produces KGM via GDP-D-glucose pyrophosphorylase and cellulose synthase-like A3. Notably, the biosynthesis of KGM requires the participation of soluble sugars such as sucrose, fructose, and glucose. Furthermore, analysis of the KGM and starch contents across 5 *A. bulbifer* germplasm resources further confirms a negative correlation between these two components [37, 38]. Combined with the KGM and starch contents of 5 germplasm resources of *A. bulbifer*, it further shows that there is a certain negative correlation between them. Corm fat content (0.57–0.7%) was markedly lower than that of oil-rich crops like peanuts [39], showed had no correlation with other quality indexes, and there was no significant difference among varieties. In addition, konjac contains a large number of active ingredients KGM, which was usually processed into healthy, low-energy and low-calorie products such as konjac tofu and konjac vermicelli in the food industry, and was used to treat obesity and make slimming products, this study provides further scientific basis for it as a health food. *A. bulbifer* corms contain amino acids related to growth and development, such as aspartic acid, glutamic acid and arginine, which play an important role in glycolysis, hormone level regulation, energy supply and nutrition regulation in vivo. In this study, corms contains 0.4% to 0.6% amino acids, which was lower than the research results [14, 16]. Comprehensive analysis, the reason may be that the physiological metabolic activities of the 5 konjac germplasm resources in this study mainly produce KGM, starch and other substances; second, the seed taro in this study was older, and the amino acid content decreases with the increase of growth years. Compared with other studies [14, 40], the ash content of konjac sample in this study was higher (6.52% to 7.73%), indicating that it contains more mineral nutrients such as calcium, magnesium, potassium and iron, which was conducive to the development of its utilization value; in addition, through the analysis of mineral nutrients, reasonable fertilization methods can be formulated and precise fertilization can be made in each growth period. In addition, there were 11.55 % to 14 % crude protein, 2.11% to 3.24% soluble sugar, 4.11% to 6.29% crude fiber and other substances in the corms of *A. bulbifer*. The composition characteristics of nutritional components of *A. bulbifer* were revealed together with other indicators, which provided scientific basis for its development and utilization.

In this study, PCA analysis was performed on 14 indicators of growth, yield and quality, among which PC1 mainly included petiole diameter, leaf width, yield and crude protein, while PC2 was dominated by KGM and viscosity. The 5 varieties could be divided into three groups. The first group was ‘YunRe1701’, which had higher plant growth, higher yield than other varieties. The corm was rich in starch, soluble sugar, crude protein, crude fiber and other nutrients, but displayed low KGM content and suboptimal viscosity. The second category was ‘YunRe1709’ and ‘YunRe1726’, both of which had strong plants, high corm yield, KGM content and viscosity in the corm were up to the standard of konjac powder. ‘YunRe1709’ and ‘YunRe1726’ demonstrated balanced nutritional and economic value. The third type was ‘YunRe1707’ and ‘YunRe1710’, which had better KGM content and comprehensive performance. Konjac can be divided into KGM type, starch type and intermediate type according to KGM and starch content, so ‘YunRe1709’ and ‘YunRe1726’ can be regarded as KGM type, ‘YunRe1707’ and ‘YunRe1710’ as intermediate type, and the KGM components in them can be deeply processed. ‘YunRe1701’ belongs to starch type, compared with other varieties, ‘YunRe1701’ contains more starch, soluble sugar, crude fiber, crude fat and crude protein, and its processed scraps can be recycled for animal feed, etc. Notably, KGM biosynthesis was regulated by genes such as *CSLA*, *MSR1*, *CSLD*, *GMPP*, *PMM* and *AGP* [37, 41, 42], and molecular breeding technology can also be used to improve it in subsequent research and cultivate excellent varieties.

Metabolomic of cultivars ‘YunRe1701’, ‘YunRe1710’, and ‘YunRe1726’ revealed a diverse array of metabolites in konjac corms. Primary metabolites, including carbohydrates, amino acids, vitamins, and lipids were abundantly detected. Additionally, these cultivars exhibited rich secondary metabolites such as amines, phenols, flavonoids, alkaloids, organoheterocyclic compounds, and phytohormones, which further explored the composition of konjac and improved its nutritional value. Metabolite analysis showed that DAMs were mainly carbohydrates and its derivatives, amino acids and its derivatives, organic acids and its derivatives, organoheterocyclic compounds and lipids. KEGG enrichment showed that DAMs were mainly enriched in metabolic pathways, biosynthesis of secondary metabolites, carbon metabolism, amino sugar and nucleotide sugar metabolism, fructose and mannose metabolism. Notably, D-Gluconic acid was the common DAMs of YunRe1710_VS_YunRe1701, YunRe1726_VS_YunRe1701 and YunRe1726_VS_YunRe1710. D-Glucaric acid is synthesized from glucose- 1-phosphate via GDP glucose pyrophosphorylase to produce GDP-D-glucose, which is subsequently converted to D-glucose, and then synthesized through gluconolactonase. Here, glucose- 1-phosphate serves as the substrate for starch synthesis, while GDP-D-glucose and D-glucose act as substrates for the synthesis of KGM and D-glucaric acid. Based on this, we propose the following hypotheses: first, the production of D-glucaric acid in the bulb may drive glucose- 1-phosphate to synthesize more GDP-D-glucose, thereby reducing starch accumulation. This could explain why ‘YunRe1710’ exhibits lower starch content compared to ‘YunRe1701’, yet demonstrates higher levels of KGM and D-glucaric acid. Second, D-Glucaric acid and KGM compete for the shared substrates GDP-D-glucose and D-glucose. This competition may account for the observation that YunRe1726 has higher KGM content than YunRe1710 but lower D-glucaric acid levels. Third, the synthesized D-glucaric acid is further metabolized into intermediates such as 6-phospho-D-gluconate, pyruvate, and D-ribulose- 5-phosphate, which participate in pathways including the pentose phosphate pathway, glycolysis/gluconeogenesis, and pntose and glucuronate interconversions. Given the complexity of these metabolic interactions, advanced molecular biology techniques (such as transcriptomic analysis and real-time fluorescence quantitative PCR) are required to further elucidate the chemical mechanisms governing starch, KGM, and D-glucaric acid synthesis [43].

## Conclusions

Konjac is a vital horticultural and economic crop, with *A. bulbifer* standing out for its strong stress resistance, high yield, and superior quality, demonstrating broad application prospects. This study revealed critical interactions between aboveground biomass and underground corm yield in *A. bulbifer*, providing a scientific foundation for optimizing plant architecture and enhancing productivity through improved cultivation practices, such as environmental adjustments and targeted fertilization. Furthermore, the research elucidated the correlation between KGM and starch content, alongside their underlying molecular mechanisms. These findings lay the groundwork for future exploration of KGM biosynthesis and molecular breeding strategies. PCA analysis of five *A. bulbifer* cultivars identified ‘YunRe1709’ and ‘YunRe1726’ as high-performing varieties with exceptional nutritional and economic value, positioning them as prime candidates for prioritized cultivation to bolster the sustainable development of the konjac industry. Metabolomic profiling highlighted D-gluconic acid as a key DAM across cultivars, with its biosynthetic pathway exhibiting substrate competition with KGM and starch synthesis. Subsequent studies employing modern molecular biology techniques (e.g., transcriptomics, CRISPR) will clarify these metabolic interactions, unveiling the molecular basis of quality variation in *A. bulbifer*.

## Data Availability

Data is provided within the article.
